# A comprehensive dataset of environmentally contaminated sites in the state of São Paulo in Brazil

**DOI:** 10.1038/s41597-024-03068-8

**Published:** 2024-03-02

**Authors:** Nouha Samlani, Daphne Silva Pino, Reginaldo Bertolo, Tannaz Pak

**Affiliations:** 1 Teesside University, Middlesbrough, UK; 2 Brazilian Synchrotron Light Laboratory (LNLS), Campinas, Brazil; 3 University of São Paulo, São Paulo, Brazil

**Keywords:** Environmental impact, Water resources

## Abstract

In the Brazilian state of São Paulo, contaminated sites (CSs) constitute threats to health, environment and socioeconomic situation of populations. Over the past two decades, the Environmental Agency of São Paulo (CETESB) has monitored these known CSs. This paper discusses the produced dataset through digitising the CETESB reports and making them accessible to the public in English. The dataset reports on qualitative aspects of contamination within the registered sites (e.g., contamination type and spread) and their management status. The data was extracted from CETESB reports using a machine-learning computer vision algorithm. It comprises two components: an optical character recognition (OCR) engine for text extraction and a convolutional neural network (CNN) image classifier to identify checked boxes. The digitisation was followed by harmonisation and quality assurance processes to ensure the consistency and validity of the data. Making this dataset accessible will allow future work on predictive analysis and decision-making and will inform the required policy-making to improve the management of the CSs in Brazil.

## Background & Summary

A standard and consensual definition of contaminated sites (CSs) is not available, probably because of their heterogeneous nature. Different entities define CSs differently, following their perspective. For example, the World Health Organisation (WHO) has its own public health-oriented definition^1^ and the European Environment Information and Observation Network (EIONET) has a definition that considers mainly soil contamination and the perspective of its remediation^2^. In a broader sense, and in the context of this article, CSs range from localized instances of single chemical contamination within a specific environmental component to extensive regions affected by the simultaneous contamination of soil, water, air, and the food chain due to the cumulative impact of multiple chemicals emitted by anthropogenic activities.

CSs pose hazards to sustainable life on our planet through adversely impacting human health^3,4^ and the environment^5,6^ and therefore infringing on human rights by provoking environmental health injustice^2,7^. The exposure to CS can be direct via ingestion, inhalation, skin contact, and dermal absorption^8^. Such direct exposures occur through contaminated water, air, or soil. Contamination of surface water can be monitored through direct and frequent sampling. However, in the case of pollution percolating to groundwater, establishing the extent and spread of the contamination is not as straightforward^9^. As a result, exposure to contamination where drinking water is supplied by groundwater is more probable. The resulting adverse effects on human health tend to be delayed and silent^10^. This makes monitoring health consequences and correlating those with the underlying causes difficult. Consequently, populations exposed to these toxic environments are experiencing a violation of their human rights, particularly in light of the recent acknowledgment of the right to a clean, healthy, and sustainable environment by the Human Rights Council^11,12^.

**Fig. 1:**
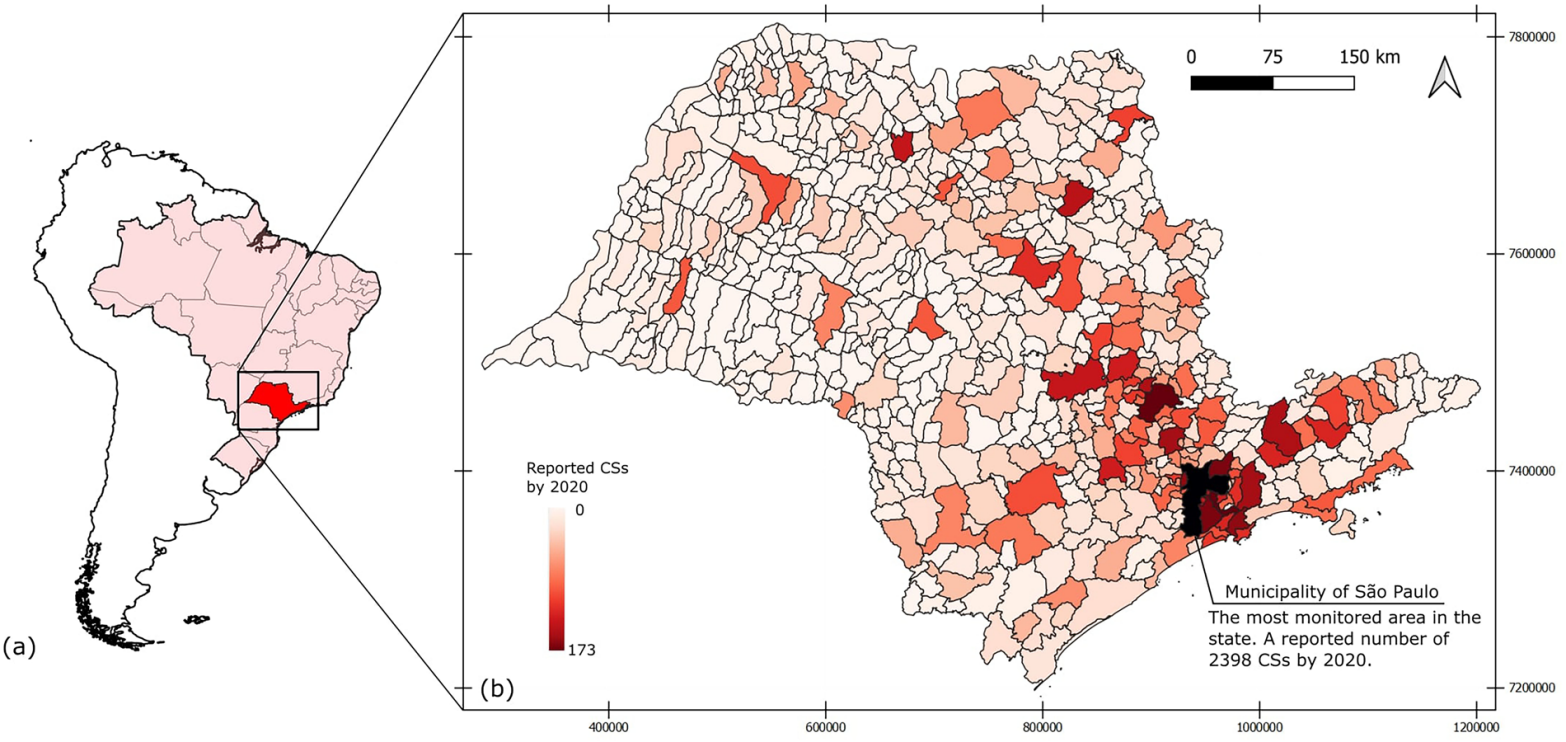
(**a**) A map of Brazil showing the location of the state of São Paulo (study area). (**b**) Distribution of the monitored contaminated sites in this state per municipalities based on the 2020 CETESB report.

CSs are reportedly growing in numbers in different countries. For example, recent research estimates that potential CSs are more than 2.5 million in Europe^8,13^ and 0.5 million in China^14^. For Brazil, the official number of CSs has not been reported, although such an estimate is required by its legal provision of soil quality^15^. There is, however, an approximate estimate of at least 8000 and 14000 CSs related to gas stations and industries, respectively, in the state of São Paulo (Fig. 1a) alone^16^, the most populous and industrialised state in the country. This number is a prediction based on the monitoring data collected by the environmental agency of São Paulo (CETESB).

**Fig. 2:**
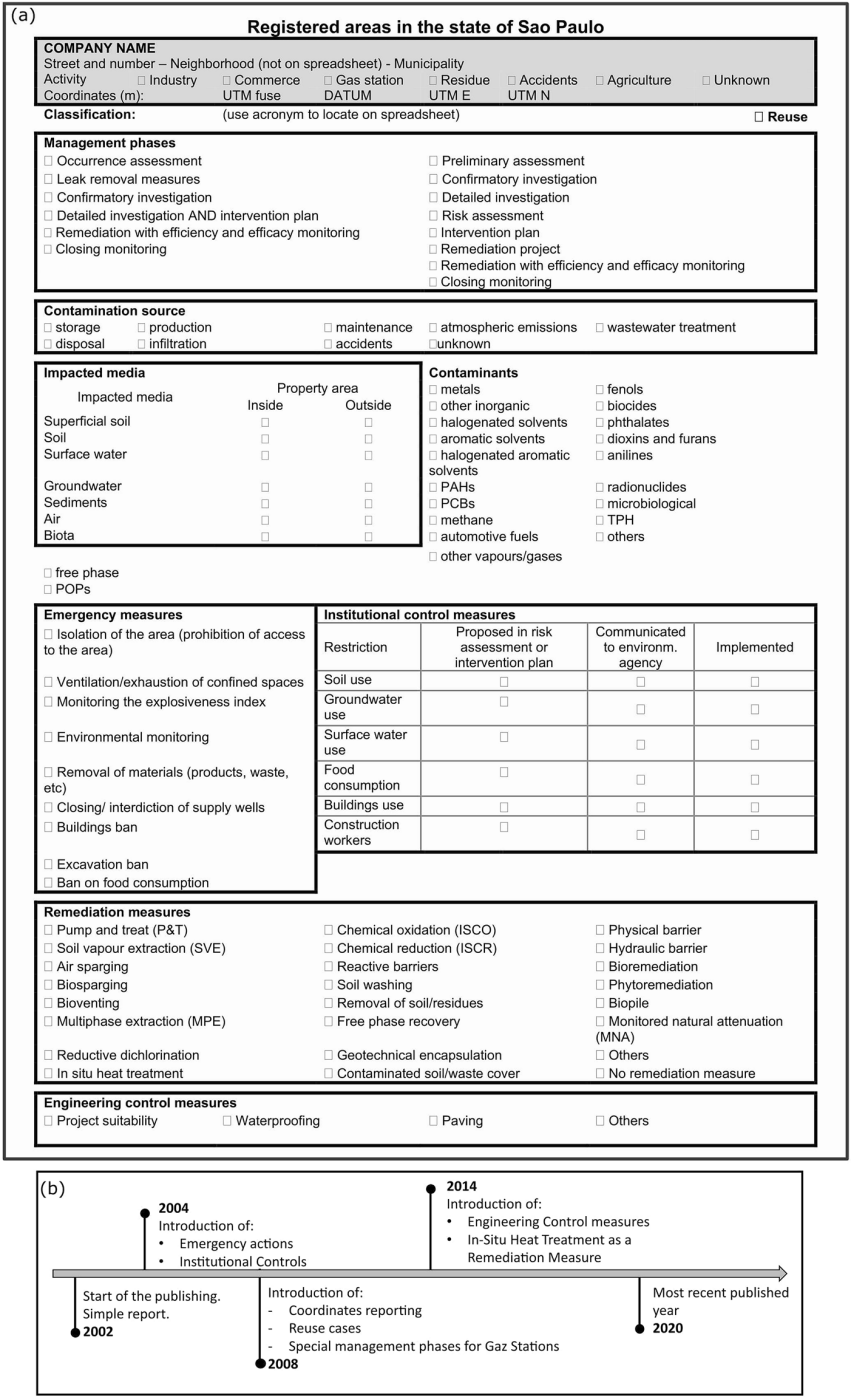
(**a**) Sample of the latest survey form version used by CETESB translated to English. (**b**) Timeline of the notable amendments to the survey form.

CETESB has been publishing monitoring data since 2002 using standardised survey forms. The data is gathered from environmental reports elaborated by companies consulting site owners. With a total of nine sections, the forms are designed to qualitatively characterize the contaminated sites considering two key aspects: the contamination and the site management. The forms contain data as text and checkboxes. Figure 2a illustrates a version that was adopted since 2014. Prior to this, the survey forms were subject to several additions and changes. These changes reflect the evolution of the monitoring and the technologies available. Introductions concern management-oriented sections such as emergency, institutional, and engineering measures. For example, an update introduced in 2014 was the addition of *in-situ* heat treatment as a remediation technology. The timeline in Fig. 2b shows the notable additions.

**Fig. 3:**
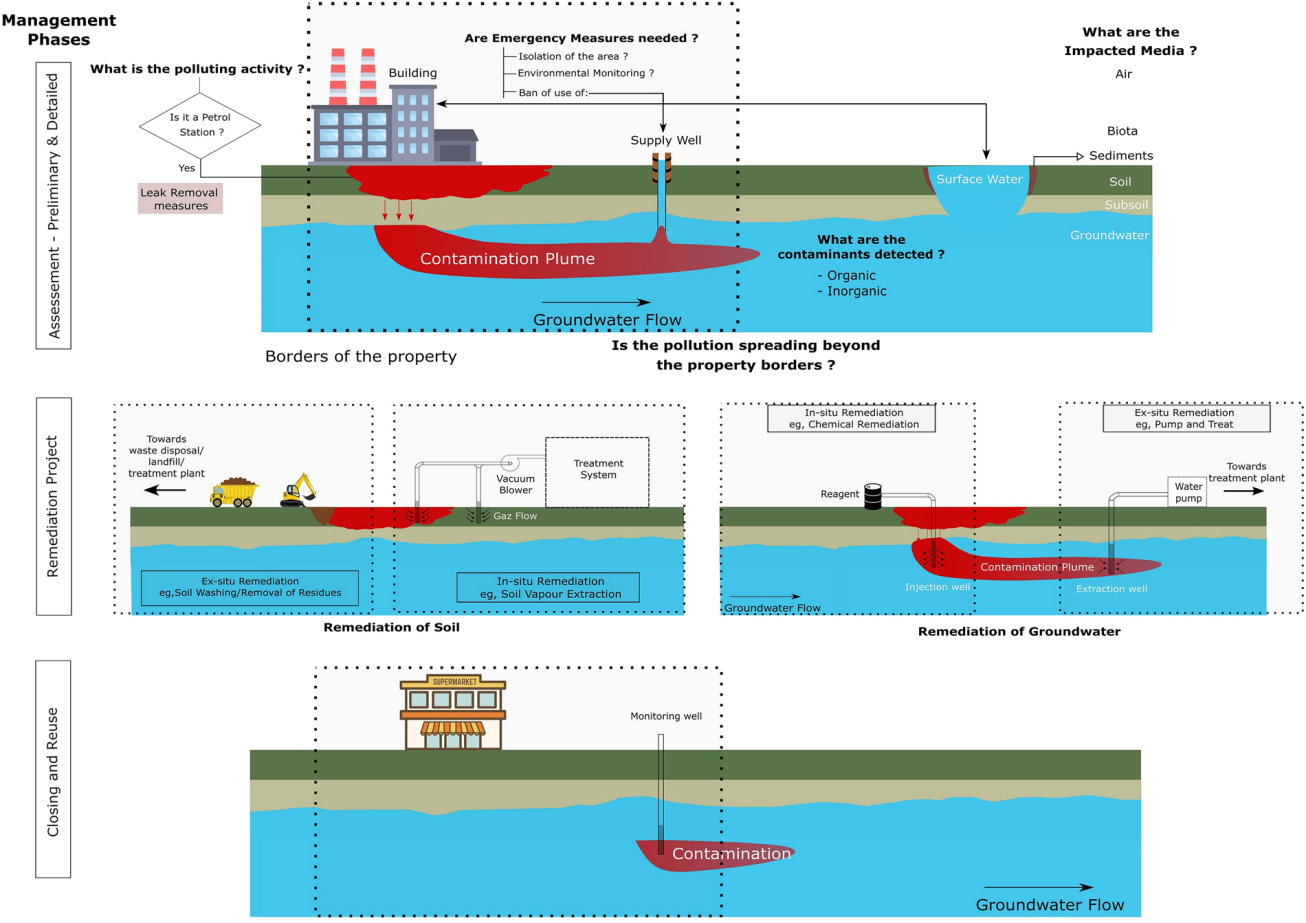
Summary of the information captured in different sections of the survey forms used by CETESB.

This dataset informs on the contamination and management of the CSs. It provides data about the contamination source, extent, and pathways affected, as well as the management and remediation processes of soil and/or groundwater. Figure 3 gives a conceptual overview of the monitoring and remediation process as well as a summary of the information captured in this dataset. The area covered in this dataset is the total extent of the state of São Paulo in Brazil. Being at the heart of the south-eastern region (Fig. 1a), the state of São Paulo is the most economically productive in the country as it produces more than half of its industrial products and an important fraction of the agricultural products^17,18^. In addition, this state stands out as one of the most groundwater-dependent states in the country^19^.

In recent years, research confirmed that the rising anthropogenic contamination threatens soil and groundwater resources, particularly in the urbanised and industrialized districts^20^ of the state. The high number of reported CSs in the state by 2020 (Fig. 1b) corroborates the pollution threats.

The public disclosure of the CSs monitoring and management data is required by the CONAMA resolution 420^21^. CONAMA refers to the federal agency that is the Brazilian National Environmental Council. However, to this date, only three states out of the 27 Brazilian administrative divisions are following these requirements, the leader being the state of São Paulo.

Large environmental datasets resulting from consistent surveying over time have shown to be great tools for advancing science and impacting regulations^22,23^. Unfortunately, CSs have not been the subject of such rich and robust datasets^24^. While providing monitoring data in the public domain is a valuable step towards the democratisation of information, such as in the case of São Paulo State, the lack of digitisation of these data poses an obstacle to their practical use in research and decision support.

This work addresses this gap by assembling a CSs monitoring dataset spanning over 17 years (2004–2020) on a state scale by digitising the yearly survey forms published by CETESB. To our knowledge, this could be the first open-access digital dataset for CSs in Latin America in the English language. Concretely, we extracted and harmonised the entries over the years and organised the monitoring data (collected using computer vision-based algorithms) in tabular format.

The digitisation of 17 years’ worth of data monitoring can be a heavy undertaking; therefore, it is of great value to build a dataset in a ready-to-use format and in adherence with the FAIR data principles^25^ – Findable, Accessible, Interoperable, and Reusable. In accordance with these principles, the dataset is configured to be easily findable, boasting a unique DOI and comprehensive metadata. It resides openly in a public data repository, ensuring easy access for researchers and the public. Presented in a tabular format with the “.xlsx” extension, the dataset lends itself to seamless integration with various analysis platforms and tools, ensuring interoperability. To ensure its reusability, the dataset is released under an open license, accompanied by thorough documentation detailing the data source and the intricate steps involved in its creation.

Once digitised, this dataset offers a data source for purposeful data mining applications to inform regulations and support optimized decision-making. For instance, by studying the entire dataset, conclusions can be made on the most frequently used remediation measures and, therefore, provide an overview of the remediation sector in the state. Furthermore, the average remediation period (time taken from site assessment to closure) for different contamination contexts can be computed, which would help identify time-effective remediation technologies/scenarios for specific sites. Eventually, more advanced applications of this dataset are to identify potentially contaminated sites that are not already on the register. Such studies could guide CETESB’s monitoring efforts and optimize remediation efforts, resulting in more efficient use of resources.

Finally, we believe that making this dataset available in English will draw more attention to Brazil’s pollution of soil and groundwater resources. This can energize future research projects in this area.

## Methods

### Source of data and pre-processing

The CETESB agency was the first of its kind in Brazil, established in 1968^21^. It has not only made a leap of advances within the state of São Paulo but has also largely influenced the CSs management in other Brazilian states. CETESB publishes yearly registries of monitored CSs in Portuguese through their website: https://cetesb.sp.gov.br/areas-contaminadas/relacao-de-areas-contaminadas/. At the time of article composition, the dissemination of forms had been replaced with interactive thematic dashboards. However, the forms used as input data for this work remain available for download and reuse.

**Fig. 4:**
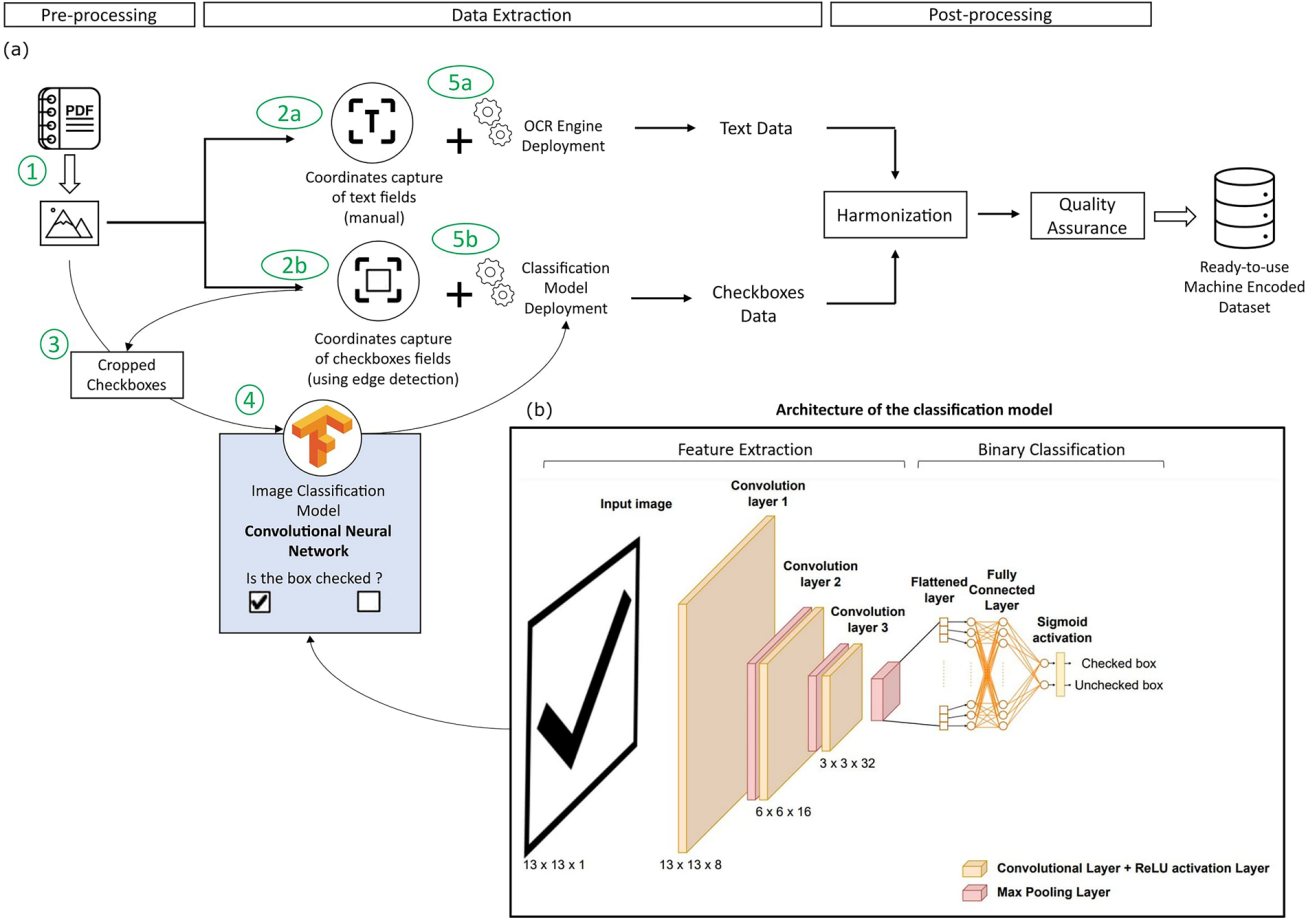
(**a**) Workflow used in the automatic digitisation of the CETESB survey forms. (**b**) Architecture of the CNN used for image classification.

This consistent monitoring spanning almost two decades has generated more than 70,000 survey forms of the type shown in Fig. 2a. While these forms are open access, they are in Portuguese and are in a non-encoded machine format presented in Portable Document Format (PDF). To prepare the dataset for data extraction, a number of pre-processing operations are required on the PDF forms. The main steps are splitting the dataset for each year into individual PDF files and converting them into images (step 1 in Fig. 4a).

### Data extraction

Automating the digitisation of the registries is necessary as this is a time and resource-consuming task if attempted manually. The solution presented in this paper is an alternative to the commercial form recognisers provided by prominent technological companies that impose paywalls and present data privacy issues. It is developed to run locally and adapted to the registry in question.

We deployed computer vision techniques to recognise both the text and the checkboxes in the survey forms. Tesseract, an open-source Optical Character Recognition (OCR) engine maintained by Google since 2005^26^, was used for text interpretation. The architecture of the fifth version of Tesseract includes a Long Short-Term Memory (LSTM) model. The LSTM model has improved Tesseract’s performance^27^ compared to its legacy model, especially in recognising diacritics. For simplification and organisation reasons, we assisted the recognition of the text by manually capturing coordinates of the usual emplacement of the text (step 2a in Fig. 4a). Then, deploying the OCR engine enabled the interpretation of the text (step 5a in Fig. 4a).

In parallel, a combination of edge detection and image classification was used for the detection of checked boxes and their classification. Edge detection was performed using a Python algorithm which enabled the detection of the bounding rectangles of the checkboxes (step 2b in Fig. 4a). After labelling the boxes with their respective field name, a value of 0 or 1 is assigned for unchecked and checked boxes, respectively. The classification was achieved using a custom-trained Convolutional Neural Network (CNN) based image classifier trained to recognise the checked and unchecked boxes (step 5b in Fig. 4a). CNNs are the most prevalent neural network models used for image classification tasks. The usual structure of a CNN includes multiple layers, such as convolutional layers, pooling layers and fully connected layers. In convolution layers, the network deploys filters to the input images to extract features for prediction. The output of each filter is called a feature map. A non-linear activation layer follows each convolutional layer. The activation function adopted is the default rectified linear activation unit (ReLU)^28,29^, which allows complex relationships in the data to be learned. Pooling layers are used to reduce the dimensionality of the feature maps and help prevent overfitting. The output of the convolutional and pooling layers is flattened into a 1D vector and then fed into one or more fully connected layers, also known as dense layers. Each neuron in the fully connected layers receives input from every element in the flattened vector, enabling the network to learn high-level representations and relationships among the extracted features. The last connected layer of a binary classifier contains a sigmoid activation function that enables the classifier to provide a probabilistic prediction. The output of the sigmoid function is a value between 0 (negative class) and 1 (positive class), which can be interpreted as the likelihood of the input image belonging to the positive class. A threshold (in this case, 0.5) is then used to determine the final class prediction. The architecture of the model deployed (Fig. 4b) comprises three convolutional layers alternating with three max-pooling layers followed by one connected layer. Table 1 summarizes the purpose of every methodology step (the green numbers in circles in Fig. 4a), the software details, the outputs, and the corresponding code files available in the GitHub repository.

### Data harmonisation

The changes in the form sections over the years have resulted in inconsistent variable names, especially for the early years. The form, as presented in this paper, is the latest version which has been in use since 2014. Therefore, the data for 2014–2020 are entered under consistent variable naming, which removes the need for harmonisation. However, in the earlier years of surveying, many sections have been subject to modifications. The most modified data fields concern the managerial components such as the classification and the management phases. The harmonisation adopted for the aforementioned sections is explained in due course in the data records.

## Data Records

The transcribed data extracted from the forms is stored in Excel files according to the publication year is available under the folder “Dataset files” in the figshare repository^30^: https://bit.ly/464nqJJ.

Each line of the Excel files contains data captured from one form. For checkbox fields, binary values are assigned: 0 for unchecked and 1 for checked. Field names are designed to be straightforward, each paired with a prefix indicating their respective sections in the form. Additionally, more detail is provided in a file named “Guide to the excel files.pdf” available within the dataset repository.

### Site identification

This section contains the name and the address of the site-occupying company/activity. The polluting activities reported are numerous and vary from industries and petrol stations to cemeteries. Starting from 2008, the coordinates of these sites have been reported in the forms to capture their exact location. Four different datums and two different fuses are used: the World Geodetic System 1984 (WGS84), the Geocentric Reference System for the Americas (SIRGAS2000), the South American Datum 1969 (SAD69), and Córrego Alegre on the 22^nd^ and 23^rd^ fuses. This difference is due to the base maps used by the different consulting companies.

### Contamination

The contamination is characterized by the type of polluting activity, the contamination source, the contaminant type, and the impacted medium. The pollution can be attributed to a discernible activity, such as industrial operations, commercial activities, petrol station facilities, or, on a less frequent basis, agricultural practices; or it can be the consequence of a spillage accident or a poor disposal or storage of a harmful residue. Petrol stations are classified separately, distinguishing them from the general commercial category. This reflects the federal resolution CONAMA n.273 of 2000 that established the guidelines for the environmental licensing of petrol stations and the prevention and control of pollution. Besides, information on the process or operation source of contamination (e.g., storage, production, disposal) leading to the contamination is specifically reported.

**Fig. 5:**
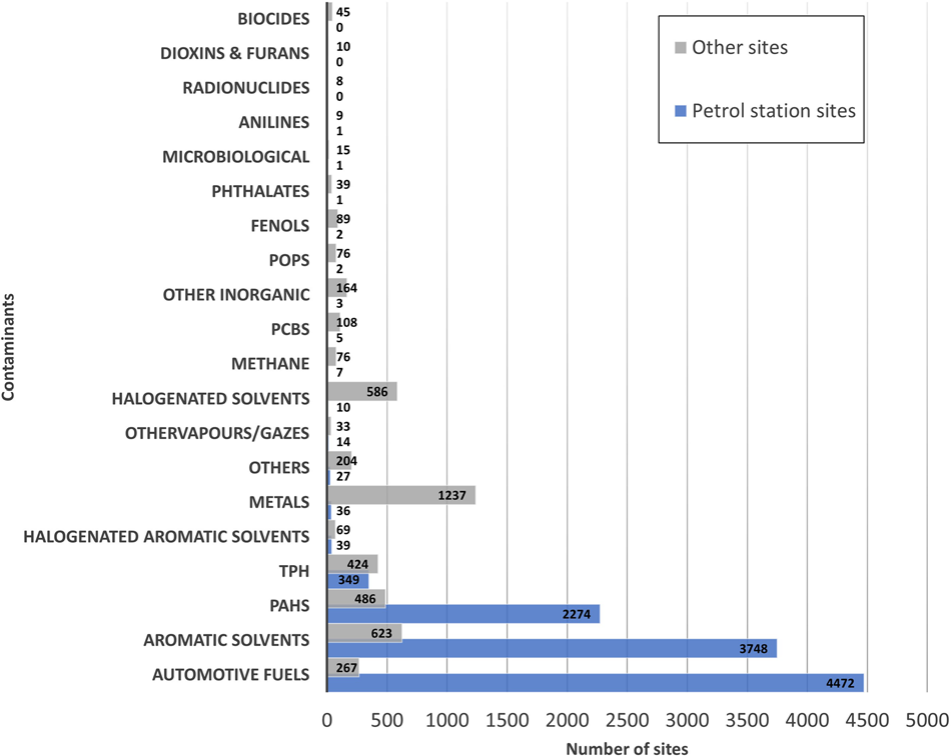
Contaminants detected in the monitored sites by the year 2020 in petrol station sites and other sites.

Several types of contaminants are monitored (Fig. 5). More than 86% of the sites by 2020 are reported to be affected by more than one type of contaminant. Predominant contaminants at petrol station sites are automotive fuels, with a detection rate of 98%, followed by aromatic solvents and polycyclic aromatic hydrocarbons (PAHs) at detection rates of 82% and 50%, respectively. In contrast, metals are identified in 64% of industrial sites, followed by halogenated and aromatic solvents, each detected in over 30% of cases.

Such an organised and yearly monitoring of CSs provides valuable insights into the key contamination pathways (e.g., soil, water, sediments, air). It also allows identifying cases where the contamination has spread in a particular medium and has reached outside the CS boundaries. This can make the remediation a more complex project as it will involve collaboration between neighbouring sites of typically different scales and financial abilities.

### Management

**Fig. 6:**
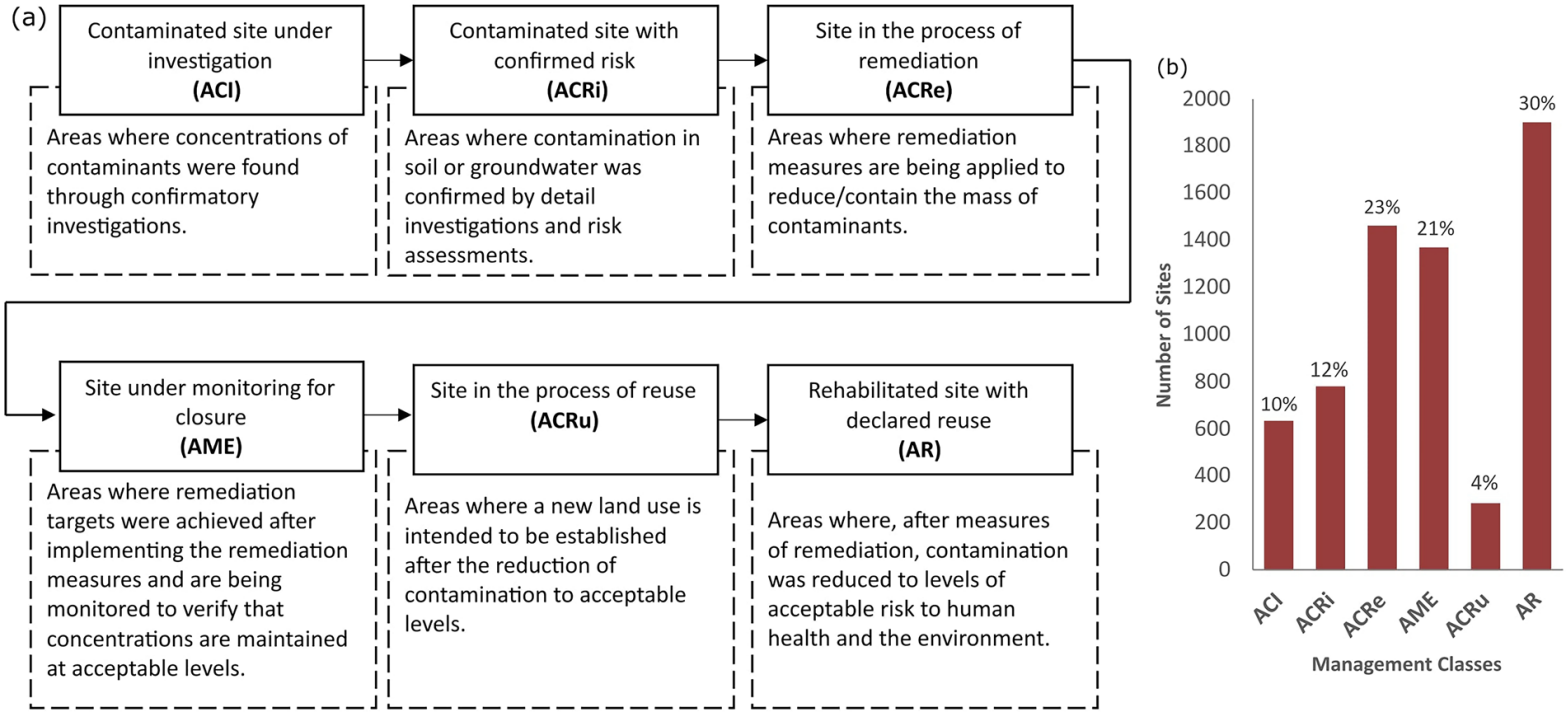
(**a**) The key management classes of the registered areas and their definitions. (**b**) Percentage of each class by the year 2020.

The classification of a site is an important aspect captured in the form and has been reported since the beginning of the monitoring program. In 2013, with Article 8 of Decree n. 59.263^31^, a standardised and more comprehensive classification of the sites was adopted. The article defines ten classes, but the six most frequent ones in the dataset are explained in chronological order in Fig. 6.

**Fig. 7:**
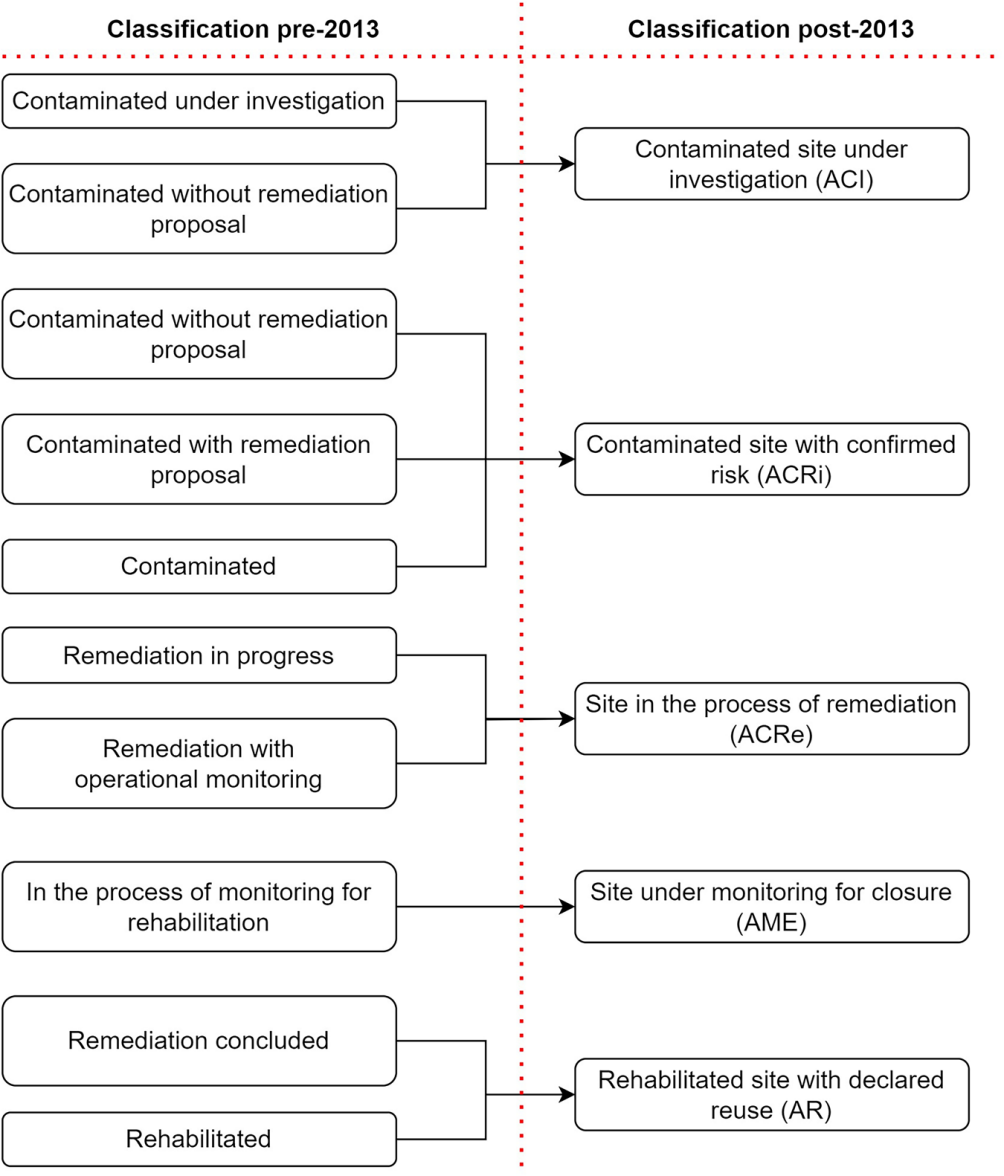
Harmonisation of pre-2013 site classification system.

Before the provisions of article 8, the classification focused on contamination detection and investigation rather than remediation. This led to detailed categories of contamination. For instance, a site could be classified as “Contaminated without remediation proposal”, “Contaminated with remediation proposal” or “Contaminated” which would all fall under the one class of “Contaminated site with confirmed risk” (ACRi) in the new classification system. Furthermore, the old classification overlooked the closure and reuse aspects as the most advanced class for a remediated site would be labelled as “Remediated”. Conversely, the new classification system aims to highlight the remediation phase, thus creating more balanced classes illustrating the whole site management process. Therefore, the entire dataset has been harmonised in this regard and is reported in the new classification system (Fig. 7).

**Fig. 8:**
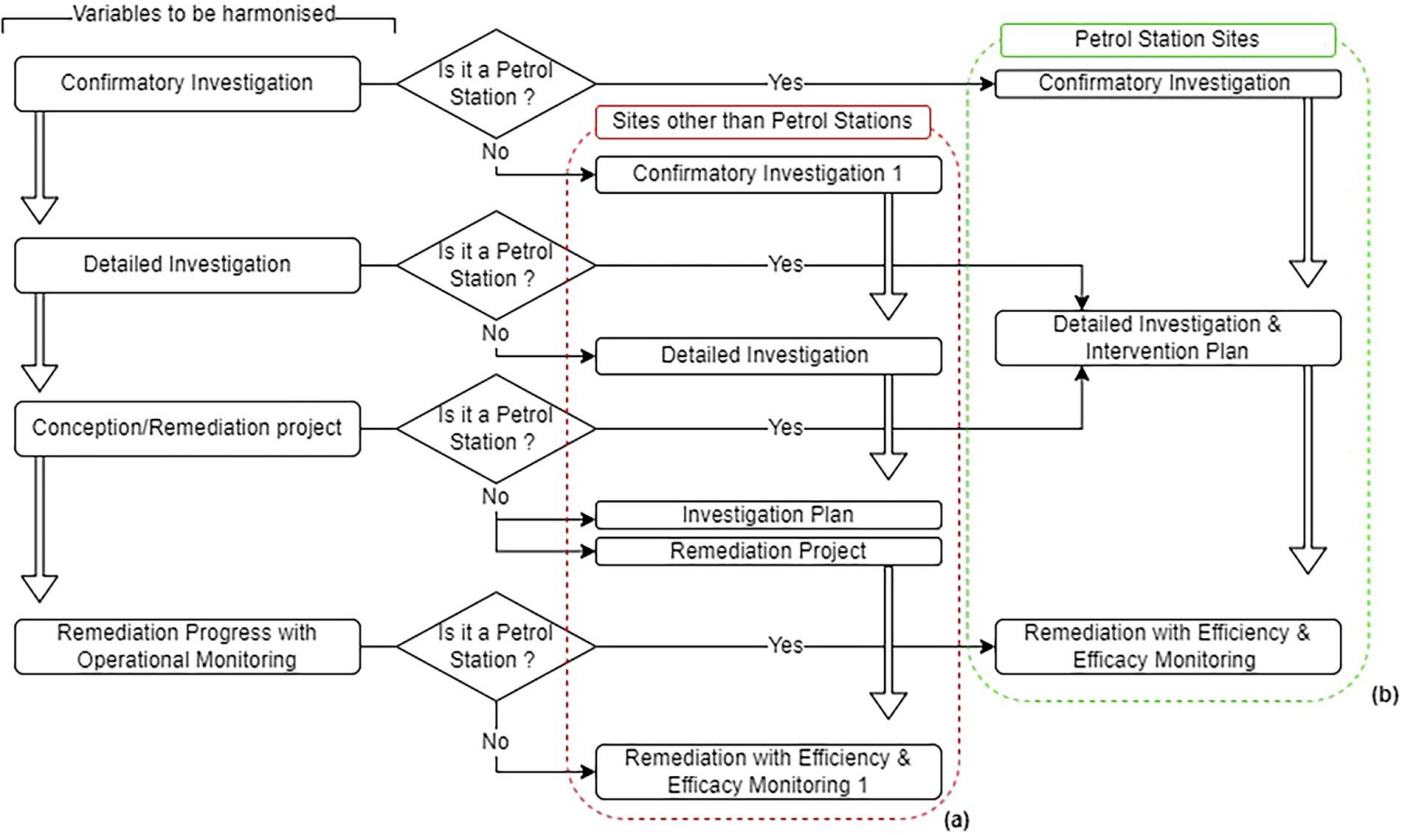
Harmonisation of pre-2008 management phases (**a**) for sites other than petrol stations and (**b**) for petrol station sites.

Before 2008, each contaminated site would go through five managerial steps: confirmatory investigation, detailed investigation, risk assessment, conceptualization/remediation project, in the process of remediation with operational monitoring. These management phases have been updated since 2008. Two notable changes were applied. First, more steps were added to the management program: a preliminary assessment as a starting step, and monitoring for closure as a final step. Second, specific managerial steps were tailored to the case of petrol stations. These include an assessment of leak occurrence and leak removal measures. For harmonisation purposes, the management phases were reported using the updated version (Fig. 8).

Intervention measures can be implemented for each case, divided into emergency, institutional and engineering actions. The measures are either proposed in a risk assessment or an intervention plan and should be communicated to the environmental agency and then implemented. Finally, the last measures to be introduced are the engineering measures that monitor the project suitability and engineering solutions applied to the soil interface (e.g., paving, waterproofing).

**Fig. 9:**
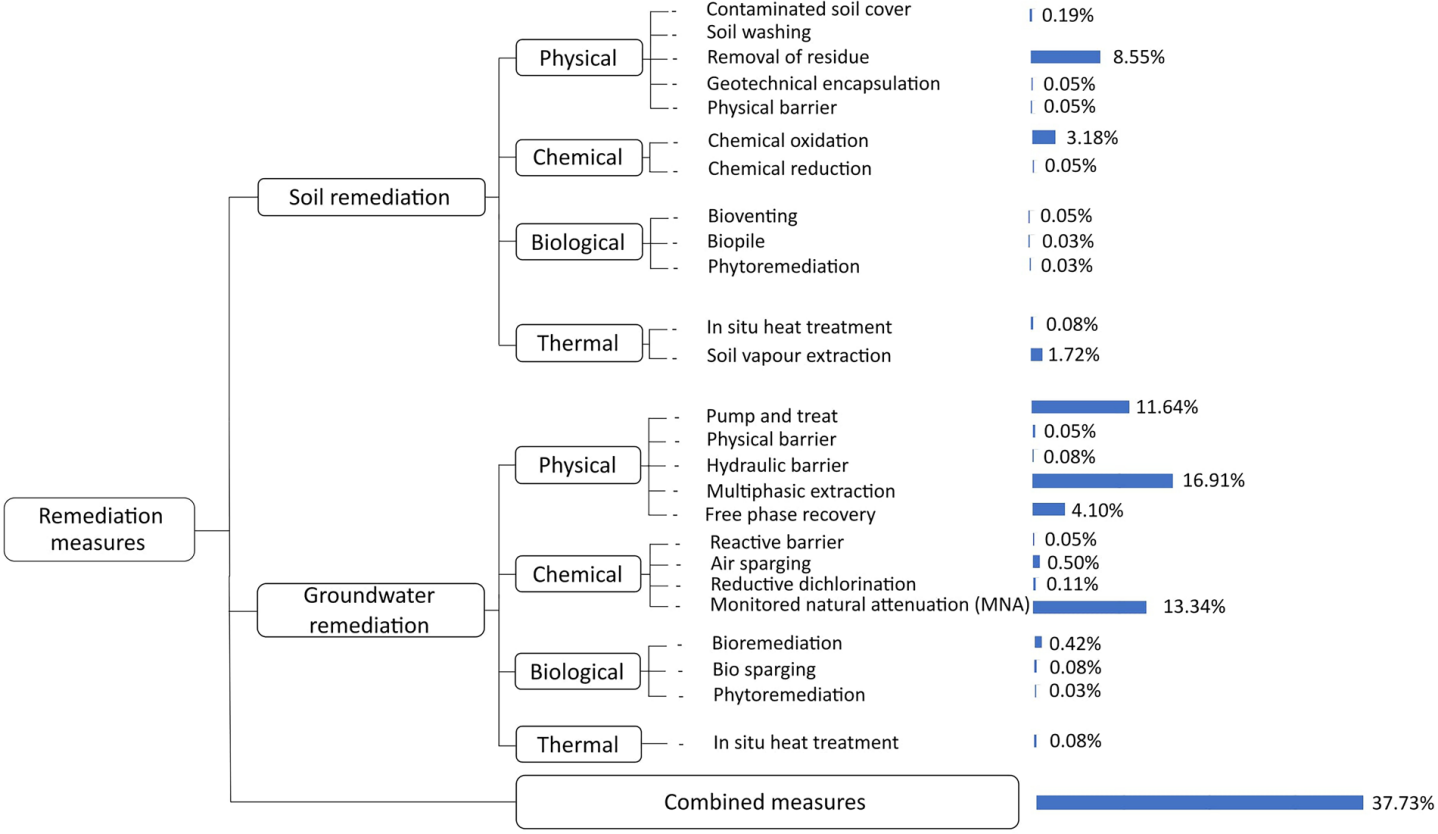
Remediation measures options in the survey form, classified by nature of the intervention, and with percentages of their implementation in the sites monitored by the year 2020.

Lastly, a total of 22 technologies are listed as options in the remediation measures sections for both soil and groundwater. Various conventional and cutting-edge techniques^32,33^ can be classified into four classes (Fig. 9): physical, chemical, biological, and thermal. Physical remediation, which entails removing or containing the pollutant, tends to rely on an *ex-situ* treatment facility or disposal site. Therefore, this type of remediation involves the additional cost of transport and/or disposal of the soil/water. However, when the treatment option is included, it offers accelerated and controlled treatment. Chemical remediation includes measures that chemically enhance the degradation by conversion or bond breaking. Bioremediation comprises techniques that use living organisms (e.g., bacteria, fungi) to break down pollutants. Phytoremediation is a specific type of bioremediation that uses plants to remove, degrade, or stabilize contaminants in soil or water. Finally, thermal remediation refers to the use of heat to volatilize or destroy pollutants. A combination of these methods is often deployed to reach rehabilitation (Fig. 9). The widely used techniques are mostly physical (e.g., pump and treat, multiphasic extraction, removal of residue) and chemical to a lesser extent (e.g., monitored natural attenuation, chemical oxidation). This is a direct consequence of the type of source of contamination.

## Technical Validation

### Text extraction validation

In the site identification section, the survey forms feature fields containing different types of text: numeric-only text used for the coordinate fields; long-form text for the company name, address, and classification fields; and short-form text for the datum and fuse fields. From preliminary trials, the errors in text recognition were specific to numbers-only and long-form texts.

The LSTM of the OCR engine returns a confidence score that reflects the performance of every recognised text. During the OCR process, the LSTM model assigns a confidence value to each recognised character. The confidence score for a recognised word or text block is then calculated by taking the average values for all characters. The higher the confidence level returned, the more accurate the recognition of the text block is likely to be.

**Fig. 10:**
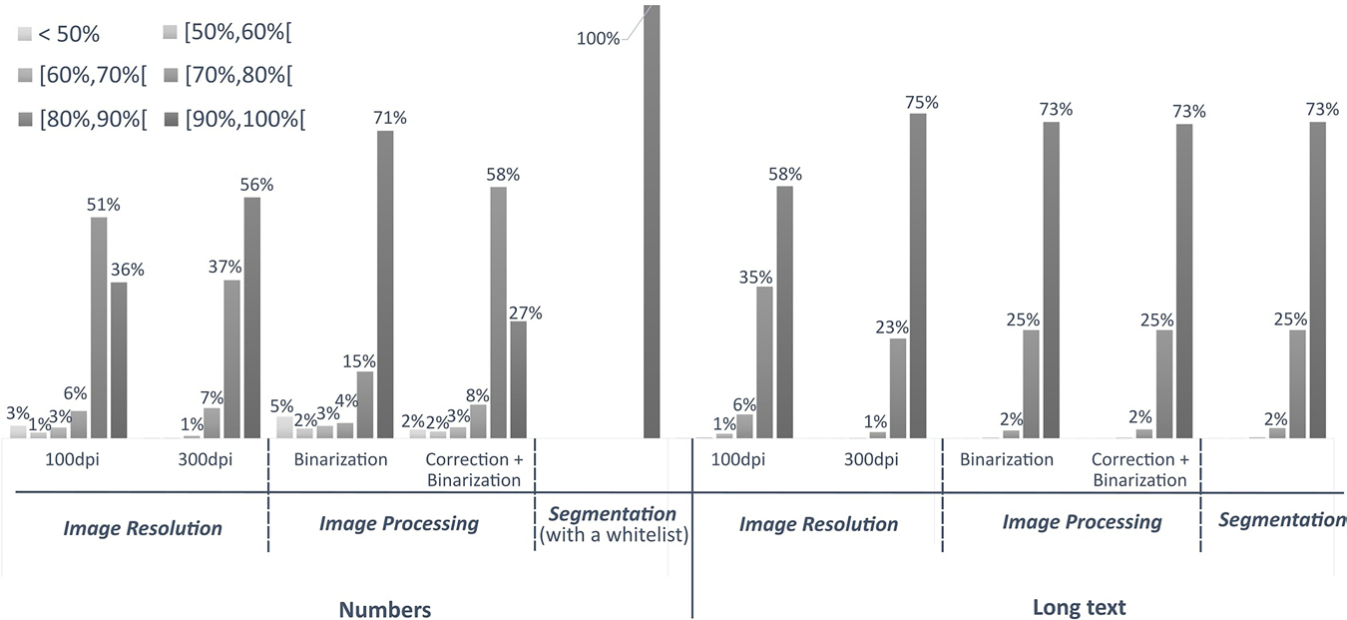
Tuning of Tesseract OCR parameters for numbers-only and long-form texts. The parameters are the image resolution, the image processing, and the segmentation modes.

There are multiple factors dictating the performance of Tesseract OCR. The resolution of the input image and the pre-processing techniques applied to it for improvement significantly impact the OCR process. Additionally, there are internal factors related to the model, such as the segmentation and engine modes. As discussed in the methods section, we fixed the engine mode to be the LSTM engine. The impact of the other parameters, i.e., image resolution, image pre-processing techniques, and segmentation modes, was investigated, and their effect is presented and compared in Fig. 10. The performances are illustrated as percentages of occurrence of ranges of confidence levels.

Improving the resolution from 100 dots per inch (dpi) to 300 dpi improves the recognition of both numbers and long texts. It particularly had a significant impact on numbers-only text, as transcription from low-resolution images yielded more than 15% of low-confidence results. Concerning the image pre-processing, we investigated the effect of binarization to remove the background noise and gamma correction to adjust image brightness. The binarization consists of converting three-channel images (RGB, Red Green Blue) to one-channel black and white images. While it did not have an evident effect on long-form text images, binarization yielded good results for numbers-only text, increasing the 90%–100% confidence range by 15%. Adding a gamma correction process prior to the binarization of images was not beneficial in either case and, therefore, was not included in the final configuration of the model. Finally, two segmentation modes (i.e., to identify and segment text regions within an image) were explored. These were the ones that treated the images as a uniform block of text or as a single text line. These segmentation modes did not have a tangible effect on the performance of the OCR. However, including a “whitelist” containing a list of numbers from 0 to 9 and decimal symbols achieved a 100% recognition rate for the numbers-only texts within the 90% to 100% confidence level.

Although the confidence score of the LSTM gives a clear insight into the model’s performance, reviewing the OCR results against ground truth data is essential. To evaluate this accuracy, the metric used is the Character Error Rate (CER)^34,35^. CER measures the percentages of incorrectly recognised characters in the OCR output relative to the total number of characters in the ground truth text. These can be caused either by substitutions, deletions, or insertions.

The OCR-recognised texts that scored less than 90% confidence level were compared against their corresponding ground truth texts. The numbers-only text has shown a very low error rate (a CER close to 0%), while the long-form text had an average CER of 1.4%. This error rate was caused by 30% deletions (of spaces), 28% insertions (of characters such as “—”, “/”, “ $”, “?”, “ |”, “.”), and 10% substitutions (most frequent example: “I” => “|”). These inconsistencies are not only easily identifiable, but they also do not cause a significant loss of information in the text. For example, the maximum CER reported for a long-form text was 18% in a case where the OCR recognised “AUTO POSTO 14 BlS LI DA.” instead of “AUTO POSTO 14 BIS LTDA.”.

In conclusion, Tesseract OCR using Portuguese training data has achieved decent results overall; hence a custom re-training of Tesseract is not necessary. However, a post-OCR manual review and data cleansing, guided by the confidence level values, is essential to guarantee data accuracy.

### Checkboxes classification validation

**Fig. 11:**
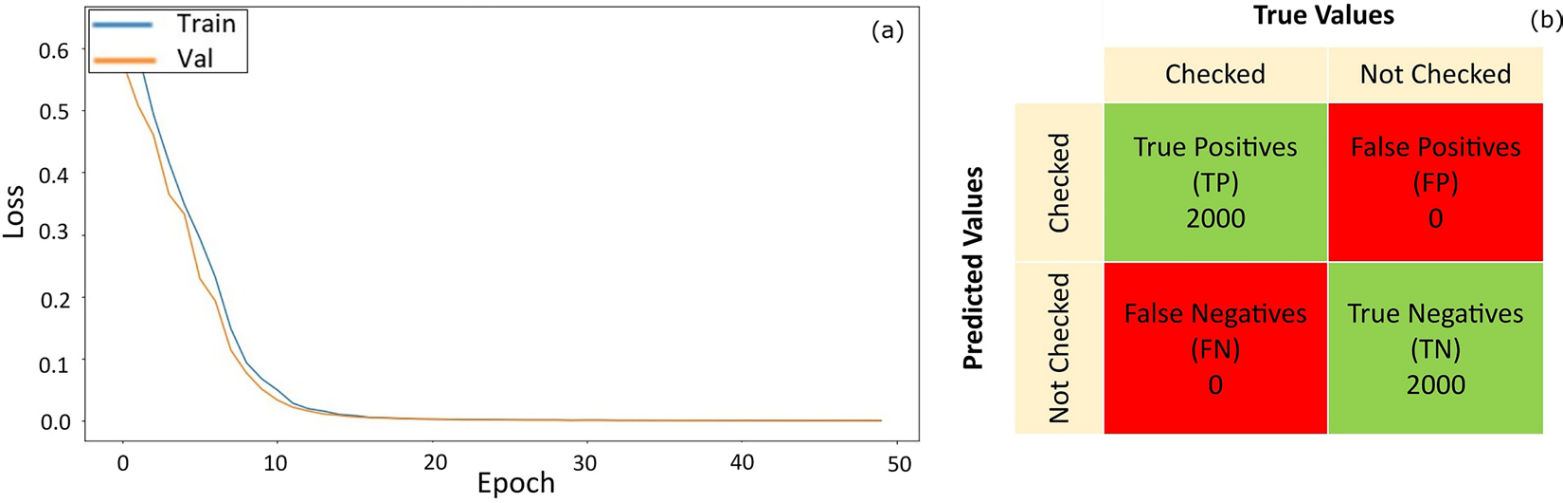
Performance of the image classifier model in (**a**) the learning phase and in (**b**) the testing phase. (**a**) shows the loss curve of the training and the validation sets. (**b**) shows the confusion matrix of the testing phase.

The image classifier was trained and validated on 4,000 checked and unchecked boxes of the year 2005, split into 80% and 20%, respectively. The two classes (checked/unchecked) are equitably represented, thus avoiding class imbalance. Figure 11a shows the decrease of the loss function over the course of training iterations or epochs. The loss function computes the distance between the algorithm output and the expected output for training and validation sets. The loss function used is the binary cross entropy^36^. The convergence of the training and validation loss functions with a minimal gap means that the model has perfectly learned the features and is able to generalise the classification for new unseen data.

The performance of the classification algorithm is measured using a confusion matrix, see Fig. 11b, a popular comprehensive performance summary for classification algorithms^37^. It is a table representing correctly classified values in both classes, true positive (TP) and true negative (TN), and the incorrectly classified values in both classes, false negative (FN) and false positive (FP). The testing phase involves applying the classification model to 4,000 new images taken from other years where the two classes are equitably represented. The results revealed that classes were correctly predicted for the entire testing set, which translates to a 100% accuracy score according to Eq. 1. This extremely high accuracy percentage is realistic because of the consistency of the checkboxes and symbol format throughout the years.

Once deployed on the totality of the forms, the model’s outputs were examined. First, the original probability values returned by the image classifier were verified. Intermediate confidence values (neither close to 0 nor close to 1) were absent, proving that the model is highly confident and gives decisive predictions. Additionally, a set of random forms was reviewed manually against ground truth data.

$$Accuracy=\frac{TP+TN}{TP+TN+FN+FP}$$

Equation 1: Accuracy formula from elements of the confusion matrix. TP: true positive, TN: true negative, FN: false negative, FP: false positive.

## Usage Notes

**Fig. 12:**
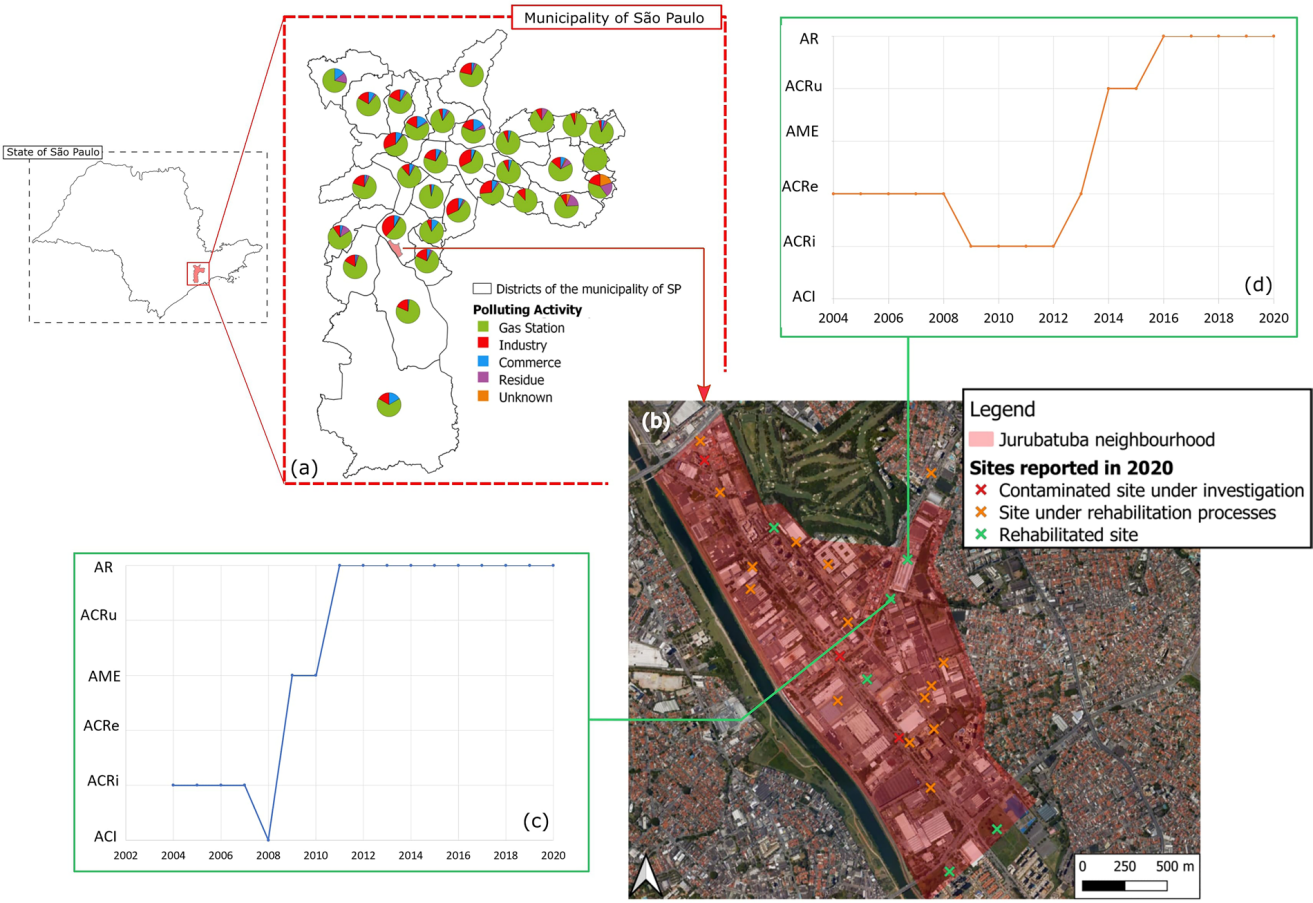
Case study on the Jurubatuba neighbourhood deploying the information in the dataset. (**a**) Zoom on the municipality of SP and the proportion of polluting activities in every district. (**b**) Spatial distribution of the reported sites in the neighbourhood. (**c,d**) Temporal evolution of the management status of two rehabilitated sites. The management phases are: ACI – Contaminated site under investigation, ACRi – Contaminated site with confirmed risk, ACRe – Site in the process of remediation, AME – Site under monitoring for closure, ACRu - Site in the process of reuse, AR – Rehabilitated site with declared use.

The georeferenced temporal data contained in this this dataset can be harnessed fully using Geographical Information Systems (GIS) for visualisation and analysis. It is useful to link this dataset to additional spatial information of administrative or environmental nature (e.g., administrative or hydrological zoning, industrial districts). For instance, in Fig. 12a, we are visualising the proportions of the polluting activities organised by districts in the municipality of São Paulo. For a more localized analysis, we are taking the example of the Jurubatuba neighbourhood which is within an industrialised district. Hydrogeological and contaminant data on this neighbourhood is discussed in the literature^38–42^. Figure 12b shows the distribution of the reported sites in the dataset in Jurubatuba in the year 2020. The sites are categorized based on their management status attributed in the year 2020. The temporal dimension encompassed by the dataset allows the visualisation and the analysis of management evolution for sites of interest (Fig. 12c,d).

**Table 1:** Summary of the programmatical steps, their corresponding code files, their purpose, and outputs.

Process Step	Name of the code file	Python Package Used	Purpose	Output
**Pre-processing**				
1a	Split pdf			Individual pdfs
1b	PDF to image	pdf2image Poppler 22.01.0	Conversion of the PDF files to images	Images in jpgs format with a defined resolution.
**Data Extraction**				
2a	Coordinate capture of text fields	Computer vision package: OpenCV 4.5.4.60	Manually capture coordinates of text emplacement	Text files with coordinates and labels of the text fields.
2b	Box detection	“Box detect” package	Automatic capture of coordinates of checkboxes rectangles	Text files with coordinates and labels of the checkboxes fields.
3	Cropped boxes	OpenCV 4.5.4.60	Generate images of checked and unchecked checkboxes	Cropped images of the checkboxes’ fields of a set of forms
4	Image Classifier	Tensorflow 2.9.2	Binary classification of checked/unchecked boxes.	Trained and validated model able to classify checked/unchecked checkboxes (.h5 file).
5a	Optical Character Recognition	Tesseract version 5 Pytesseract	Text recognition and interpretation	Excel file with transcribed machine-coded text with the score of the text recognition.
5b	Image Classification Model Deployment	Tensorflow 2.9.2	Application of the classification to the cropped boxes of each form	Excel files with transcribed binary information from the checkboxes.

## Data Availability

The code files used to extract the dataset are available in the following Github repository: https://github.com/nsamlani/Code_Digitization_CETESB. The forms have changed in content and in layout over the years. Therefore, we include the bounding box coordinates for the text and the checkboxes every year (grouped text files named “bounding_boxes_text.txt” and “bounding_boxes_checkboxes.txt” for text and checkboxes, respectively). This information needs to be included in the corresponding codes to allow the reproduction of the outputs presented in this dataset.
